# The challenges of COVID-19 testing in Africa: the Ethiopian experience

**DOI:** 10.11604/pamj.2021.38.6.26902

**Published:** 2021-01-05

**Authors:** Andargachew Mulu, Amsalu Bekele, Alemseged Abdissa, Taye Tolera Balcha, Meseret Habtamu, Adane Mihret, Dawit Hailu Alemayehu, Getachew Tesfaye Beyene, Abebe Genetu Bayih

**Affiliations:** 1 Aramuar Hansen Research Institute, Addis Ababa, Ethiopia,; 2 College of Health Sciences, Addis Ababa University, Addis Ababa, Ethiopia,; 3 Clinical Infection Medicine, Department of Translational Medicine, Lund University, Malmö, Sweden

**Keywords:** SARS Cov-2, COVID-19, diagnosis, challenges, Ethiopia

## Abstract

Novel coronavirus disease (COVID-19) is spreading rapidly and creating a huge economic, social and public health challenge worldwide. Although currently an effective vaccine is ready, its distribution is limited, and hence the only currently available lever to reduce transmission is to identify and isolate individuals who are contagious. Thus, testing for SARS CoV-2 has a paramount importance. However, testing in many African countries including Ethiopia has multidimensional growing challenges. Here, we tried to identify, categorize and summarize the challenges of COVID-19 testing in Africa from Ethiopian experience.

## Opinion

Novel coronavirus disease (COVID-19) is spreading rapidly and creating a huge economic, social, and public health challenge worldwide [1]. Laboratory testing has a critical role in isolating the patient, break the transmission cycle. As of December 13, 2020, Ethiopia has conducted 1,706,321 tests since the beginning of the pandemic, according to the Federal Ministry of Health of Ethiopia (FMOH). Initially, the country was sending samples to South Africa to get the test done. The country has slowly established a testing facility at the national reference lab and later on expanded to other labs. Yet the national testing capacity is very low in relation to over a 110,000,000-population size, and the facilities concentrate mainly in major cities and university laboratories. However, this has now improved with significant development, and reached 72 laboratories with a daily capacity of 13, 944 tests. In this opinion we will showcase point-by-point the challenges we face in terms of trained human capital, infrastructure, testing kit availability, logistics constraints, etc. as a resource limited country.

One of the critical challenges we are facing is the inadequate well-trained human capital in terms of sample collection and delivery, testing and test result dispatching: it is well known that quality of sample has a significant impact in the diagnostic accuracy of any diseases [2]. At the moment, nasopharynx and/or throat swabs are used to detect SARS CoV-2 nucleic acid in Ethiopia. Here we have a great concern on the quality of samples because of lack of adequate training on how to collect good quality nasopharynx and/or throat samples, or for patient intolerance during sample collection [3]. Once collected, there are possible delays in delivering samples to testing laboratories. Although samples are stable for specified period, maintaining cold chain system is not always possible [2]. This could be a growing challenge in Ethiopia, particularly in peripheral sites weakly equipped with the laboratory equipment and could cause extended turnaround time. To identify COVID-19 cases, the only approved laboratory diagnostic approach in Ethiopia is the detection of SARS CoV-2 RNA, as this test is considered the most accurate. However, there are multiple factors affecting wide scale implementation of this test. Such as lack of trained man power to run the tests, and shortage of certified biomedical engineers capable of calibration and maintenance of biosafety and laboratory equipment are additional challenges. Besides, engaging laboratory professionals to work with samples which potentially contain the infectious virus is not a simple task. Collection of patient clinical data and sociodemographic information is manual, and electronic or web-based data collection has not been fully in use. Hence, there has been a duplication of effort in transcribing what was written at time of specimen collection and sample delivery to testing centres. Such practice is prone to human error and leads to unnecessary delay in result delivery.

Our second challenge is lack of infrastructure: health infrastructure in particular laboratory facilities with trained personnel, is very limited in sub-Saharan African countries [4]. According to WHO, a non-propagative SARS CoV-2 diagnosis should be done in bio-safety class II level-P2 laboratory [2]. However, the number of laboratories with bio-safety class II facilities are very limited in the country. Besides, most hospitals and diagnostic centers are not equipped well to handle the massive inflow samples. If the rising trend of new cases continues as it appears now, it is likely that they will soon overwhelm the limited health and laboratory infrastructures within the country. The third challenge is logistics constraints: almost all consumables for diagnosis and the test kit itself depend on the international market under the national import permit and regulations. To get minor but vital items in the local market is difficult. As a result, the Ethiopian Food and Drug Authority (EFDA) and other agencies are practicing substantially fast-tracked approval process of Covid-19 related products, yet each process of procurement should pass through the existing legal procedures (getting hard currency, shipment, certification, EFDA registration, and customs clearance) which take longer than one´s expectations.

Lack of reference laboratories is also another challenge: the currently available nucleic acid-based tests for SARS CoV-2 can miss 30-40% of those actually infected [5] because of several reasons, including individual variation and assay sensitivity. This is mainly true for samples with viral copy number closer to the detection limit of the assay. Along with testing, there is also some sort of subjectivity in setting cycle threshold, and thus standardization of testing protocol is critical. Therefore, in the presence of such uncertainties, reference laboratories are required to evaluate testing laboratories through external quality assurance programs and fill gaps.

The fifth challenge is cultural values: health seeking behaviour, the concept to disease origin and dynamics in relation to scientific knowledge in African population is low [6] As a result, people might prefer to visit religious and traditional healers than modern health care facilities. This could limit the number of individuals to avail themselves to health facilities, and impact testing of COVID-19. Furthermore, lack of work ethics of professionals could also be considered as a growing challenge related to COVID-19 risk exposure. This has been revealed by unrealistic and irrational panic, reluctance, and presentation of vague reason not to work in SARS-CoV-2 laboratories. As a result, managing fear and stigma associated with the disease are other challenges related to cultural and historical issues. In particular, stigmatization of the ill is an enormous challenge. It is a very recent memory that victims of HIV/AIDS have been heavily stigmatized across the country. As a result, individuals may become reluctant to undergo testing if they fear or other reprisals from their communities.

Last, we also have challenges from the lack of data and weak statistical capacity: the capacity of African countries including Ethiopia in addressing healthcare challenges remains hindered by a lack of data coverage, stemming from weak statistical capacity despite its importance for evidence-based decision-making and policy formulation. Authorities have inadequate access to and use of data and have the problem of using latest statistical methodologies. This represents a significant challenge for the timely production of quality data, which is the most crucial in times of epidemic emergency [7].

In conclusion, the Ethiopian government at various levels is showing high commitment to contain the pandemic before it causes significant damage to the community. The FMOH has prepared national guidelines for disease prevention, patient´s management, and diagnosis to contain the pandemic. Standardized approaches and expanding of laboratory facilities is also ongoing as one of priority areas to speed up testing. However, widespread testing seems impossible for Ethiopia unless all barriers and bottlenecks are critically considered and managed properly.

Therefore, we recommend the followings points for health authorities to take immediate actions: 1) They should enhance integrated capacity building both in human capital at various levels of the process and infrastructure development; 2) Technology based data collection system, tracking and communication system needs to be strengthen; 3) They should strengthen emergency use authorization without compromising quality; 4) Expanding testing facilities and encouraging the use of alternative testing methods such as pooling both at direct biological samples and nucleic acid (RNA) shall be started; 5) Establishing reference laboratory which design, implement, and monitor both internal and external quality control tests shall be enhanced; 6) Rapid adoption and validation of point of care tests for diagnosis of COVID-19 in our country shall be encouraged; 7) Integration of SARS-CoV-2 diagnosis with existing platform which are already widely used for TB & HIV testing shall be implemented; 8) Looking for more funds and support from international agencies and donors to support establishment of testing site and maintain testing service without interruption shall be enforced.
